# High-throughput sequencing-based analysis of the composition and diversity of endophytic bacteria community in tubers of *Gastrodia elata* f.glauca

**DOI:** 10.3389/fmicb.2022.1092552

**Published:** 2023-01-17

**Authors:** Heng Zheng, Peng Zhang, Jing Qin, Jiani Guo, Jun Deng

**Affiliations:** ^1^Emergency Department of Hubei Third People's Hospital Affiliated to Jianghan University, Wuhan, Hubei Province, China; ^2^School of Resources and Environmental Engineering, Wuhan University of Technology, Wuhan, China; ^3^Research Center for Ecology, College of Science, Tibet University, Lhasa, China; ^4^Wuchang District Shouyilu Street Community Health Service Center, Wuhan, Hubei Province, China

**Keywords:** *G* *astrodia elata*, high-throughput sequencing, endophytic bacteria, diversity, composition

## Abstract

*Gastrodia elata* f.glauca (*G. elata*) is a commonly used Chinese Medicinal Materials with great medicinal value. The medicinal plant and its endophytic bacteria are a symbiotic whole, and the endophytic bacteria are rich in species, and their metabolites are a treasure trove of natural compounds. However, there is a relative lack of analysis on the diversity, flora composition and network interactions of the endophytic bacteria of *G. elata*. In this study, high-throughput sequencing technology based on the Illumina Miseq platform was used to reveal the core microbiota by examining the diversity and community structures of tuber endophytic bacteria in *G. elata* grown under different regions and exploring the effect of region on its endophytic bacteria. Here, 1,265 endophytic ASVs were found to coexist with *G. elata* tuber in Guizhou and Hubei. At the phylum level, the dominant phyla were Proteobacteria, Actinobacteria and Acdobacteriota. At the family level, the dominant family were Comamonadaceae, Nocardicaece, Xanthobacteraceae, and Burkholderiaceae. At the genus level, *Delftia* and *Rhodococcus* were represented the core microbiota in *G. elata* tuber, which served as the dominant genera that coexisted in all samples tested. Moreover, we found that the beta diversity of endophytic bacteria in *G. elata* tuber was higher level in the Guizhou region than Hubei region. Overall, this study results to provide a reference for screening active strains and interaction between plants and endophytic bacteria.

## 1. Introduction

Plant Endophytic bacteria are important microbial-plant symbionts and microbial resource that generally exists in healthy plant tissues, which is good for plants (Wang et al., 2019, 2021; Wu et al., 2020). Some studies have found that there are a large number of endophytic bacteria that can settle in the internal tissues of host plants and form a series of mutually beneficial symbiotic relationships (Hassani et al., 2018; Aswani et al., 2020; Li et al., 2020). Plant endophytes not only promote plant growth, but also have potential application prospects in medicine, agriculture, industry and other fields (Aswani et al., 2020; Wang et al., 2021). And the establishment of plant endophyte diversity and community structure is closely related to plant varieties, genotypes, growth environment, geographical location and other factors (Rangjaroen et al., 2019). The plant endophyte is seemed to be an important determinant of plant health and productivity, and has attracted extensive attention worldwide in recent years as a subject of scientific and commercial interest (Gaiero et al., 2013; Xia et al., 2016; Wang et al., 2021). The tuber of *G. elata*, as an important part of human nutrition and drug, which are rich in microbial resources (Steinbrecher and Leubner-Metzger, 2016).

*Gastrodia elata* f.glauca is commonly called “Tian ma” in Chinese and mainly distributed in the mountainous areas of eastern Asia (Howes et al., 2017), which is a perennial parasitic herb belonging to the Orchidaceae family, to treat headache, migraine, dizziness, epilepsy, infantile convulsion, tetany and other diseases, and it is widely used in clinical practice (Xu et al., 1998; Tsai et al., 2016; Hu et al., 2019). Up to now, there is no report on the composition and diversity of endophytic bacteria community in *G. elata*. Several recent studies have shown that the endophytic microbial community associated with wild plant species may play an important role in disease resistance (Pérez-Jaramillo et al., 2016; Tian et al., 2017, 2020; Wang et al., 2019). Some studies have shown that the predominant phylum of endophytic bacteria in rice seeds were Proteobacteria, Actinobacteria and Firmicutes phyla (Zhang et al., 2018; Liu et al., 2019), the Aspergillus, Thicket and Cysticercus was dominant in different varieties of cassava (*Manihot esculenta* Crantz; Li et al., 2020), is the same true in *G. elata*? Exploring these problems is of great significance for further development and utilization of *G. elata*.

However, the composition and diversity of endophytic bacteria community in *G. elata* has not yet been studied. The core microbiota of the *G. elata* tuber and their diversity level is not clear. Here, we studied the diversity and composition of the endophytic bacteria community in six *G. elata* tuber samples under two regions and aimed to discover the core microbiota of the *G. elata* tubers and the changes of the community composition with the different regions. In this study, the community structure, core microbiota and network interaction of endophytic bacteria in *G. elata* in Hubei and Guizhou regions were analyzed to provide a reference for screening active strains and interaction between plants and endophytes.

## 2. Materials and methods

### 2.1. Sample collection and treatment

In total, six samples of *G. elata* tubers were collected from Wu Feng in Hu Bei and Da Fang in Gui zhou (Table 1) in 2022, and *G. elata* was identified by using the Flora Reipublicae Popularis Sinicae (Flora of China). During sampling, 3 healthy tubers of *G. elata* were selected and collected. Subsequently, the samples were loaded into sterile sampling bags, marked, placed in the car refrigerator at 4°C, and processed within 24 h. The samples latitude and longitude coordinates were used the World Geodetic System, 1984 (WGS-84) and were recorded using a hand-held GPS unit (Etrex 221x, Garmin, CH.).

**Table 1:** Sample location information.

**Sample id**	**Longitude (°E)**	**Latitude (°N)**	**Altitude (m)**	**GPS coordinates**	**Date**
GZ1	110.3741	30.2389	1,810	WGS-84	2022.4.05
GZ2	110.3741	30.2389	1,810	WGS-84	2022.4.05
GZ3	110.3741	30.2389	1,810	WGS-84	2022.4.05
HB1	106.6827	27.1520	815	WGS-84	2022.4.15
HB2	106.6827	27.1520	815	WGS-84	2022.4.15
HB3	106.6827	27.1520	815	WGS-84	2022.4.15

Take the sample of tubers of *G. elata*, rinse the sample with tap water, and place the sample in the ultra-clean table for surface sterilization. The specific method is as follows: Soak in 75% alcohol for 3 min, rinse with prepared sterile water for 3–5 times, soak in 5% NaClO for 3 min, rinse with prepared sterile water for 3–5 times, soak in 75% alcohol for 2 min, rinse with prepared sterile water for 3–5 times, take the last prepared sterile water to coat the plate, test the surface disinfection effect. The surface sterilized tubers of *G. elata, respectively,* cut into small pieces and put into sterile 2 ml centrifuge tubes and store at −80°C until used.

### 2.2. DNA extraction and high-throughput sequencing

The sample DNA was extracted from the filter membranes using a Power DNA Isolation Kit (Qiagen, Germantown, MD, United States) according to the manufacturer’s protocol, and DNA quality was checked using 1% agarose gel electrophoresis. DNA concentration and purity were determined with a NanoDrop 2000 spectrophotometer (Thermo Fisher Scientific, Wilmington, DE, United States). Next, the 16S rDNA V3-V4 hypervariable region was PCR-amplified using the following primers: 799F (AACMGGATTAGATACCCKG) and 1392R (ACGGGCGGTGTGTRC). All of the thermocycling steps were as follows: 5 min at 95°C; 20 cycles of 45 s at 95°C, 30 s at 57°C, and 30 s at 72°C. The amplified products were purified and mixed in equivalent amounts PCR products were sequenced using the PE250 strategy on the Illumina Miseq2500 platform by Majorbio (Shanghai, China).

### 2.3. Bioinformatics and statistical analysis

**Figure 1:**
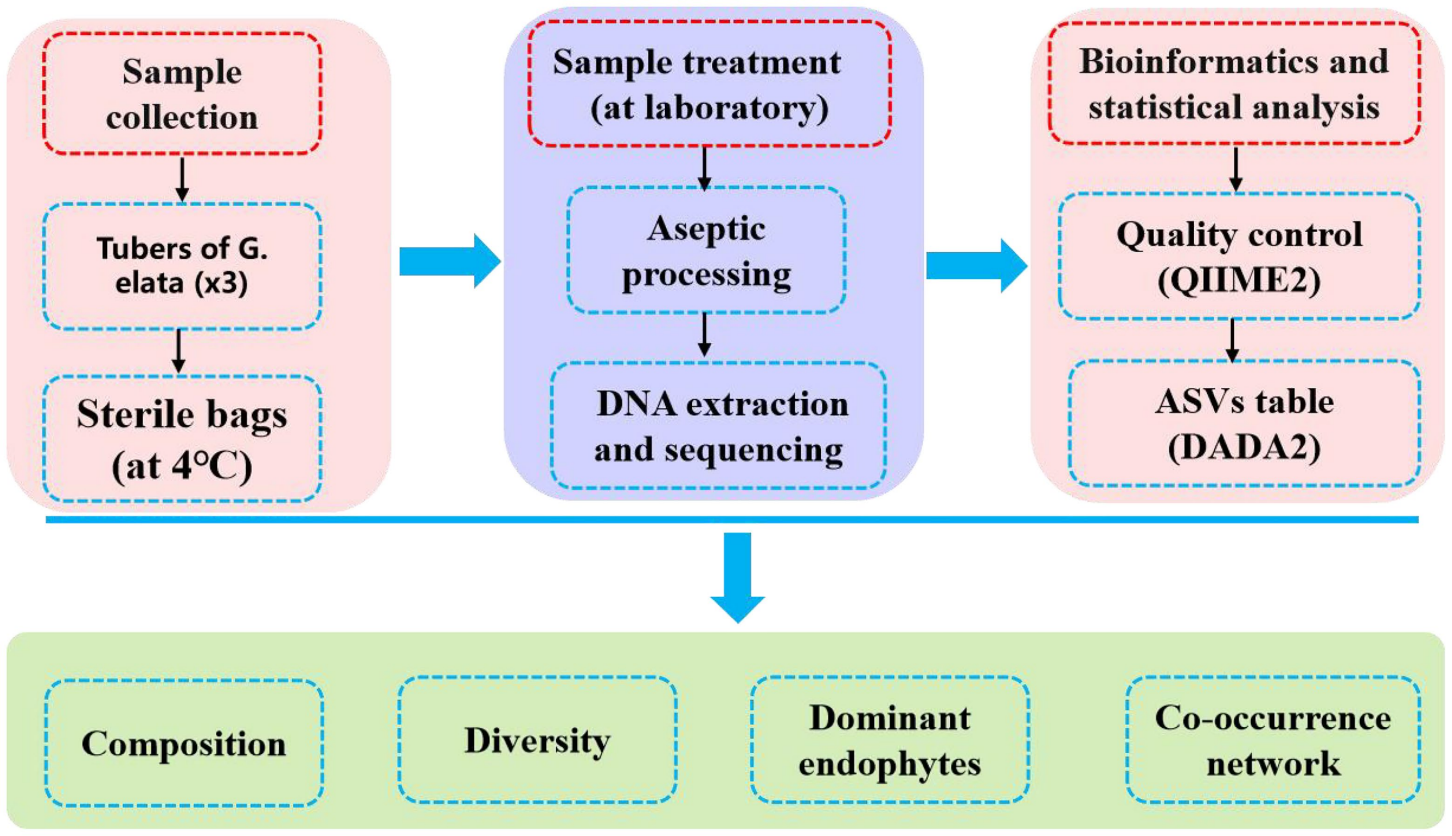
Study basic framework diagram.

After quality filtering the raw data, high quality clean data was received for subsequent analysis. The clean data were demultiplexed separately by their unique barcodes. A standard denoising pipeline was used to obtain the amplicon sequence variants (ASVs) were obtained using the DADA2 plug-in in QIIME2 software (version 2022.8; Bokulich et al., 2018; Bolyen et al., 2019), after which an ASV abundance table was constructed. The ASVs were annotated using the SILVA database (version 138; Quast et al., 2013). Low-abundance ASVs (<10 reads) were removed. Three replicates were used to reduce the sampling bias. The ASV table was then rarefied to 40,000 reads per sample for downstream analysis. Alpha diversity indices of endophytic bacteria communities, including Richness index, Shannon-Wiener diversity index, Chao1 index, ACE index, Good’s coverage index and Simpson dominance index, were calculated using the “vegan” package in R software (version 4.1.1). Principal coordinate analysis (PCoA) and Multiple samples Non-metric multidimensional scaling (NMDS) were performed based on the Bray–Curtis distance using the “vegan” and “ggplot2” package in R software. Co-occurrence patterns of endophytic bacteria communities were constructed based on Spearman’s rank correlation coefficients. Co-occurrence events were identified as statistically robust correlations (*|R|* > 0.6, *p* < 0.05) and the co-occurrence network was visualized in Gephi (version 0.9.6). The basic skeleton diagram is shown in Figure 1.

## 3. Results

### 3.1. High throughput sequencing statistics of endophytic bacteria communities in *Gastrodia elata* f.glauca

**Figure 2:**
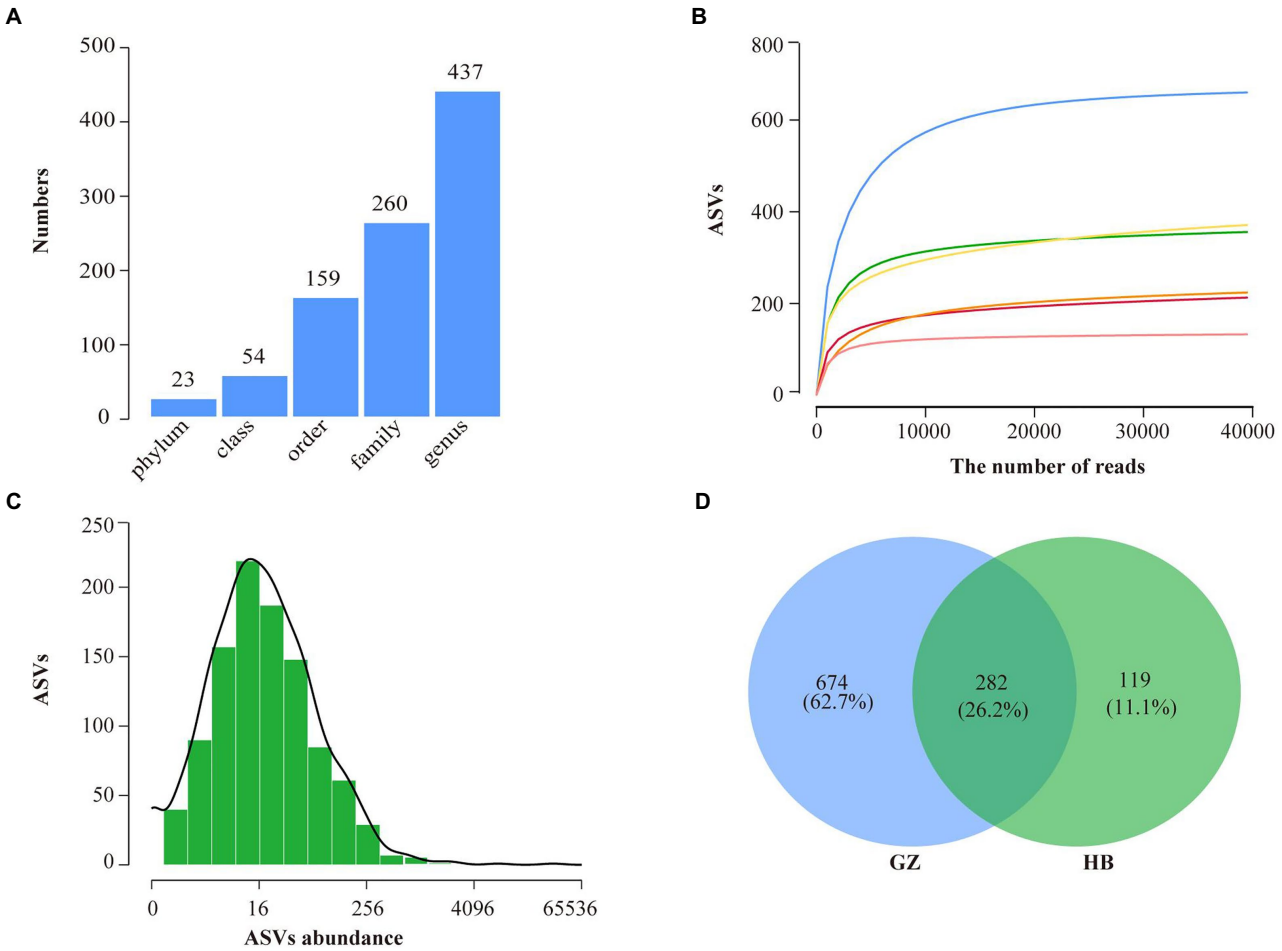
High throughput sequencing statistics and rank abundance curves. **(A)** ASV classification statistics. **(B)** ASV rarefaction curves. **(C)** Distribution of ASVs abundance of bacteria. **(D)** Venn diagram showing the number of shared and specific ASVs for each group.

A total of 280,523 raw sequences were obtained by high-throughput sequencing. After quality control, 260,543 high-quality sequences were acquired from 6 samples of endophytic bacteria in *G. elata*, which were grouped into 1,265 ASVs were identified belonging to 23 phyla, 54 classes, 159 orders, 260 families, and 437 genera (Figure 2A). Rarefaction curves indicated that most of the diversity could be covered by the resampling depth of 40,000 reads (Figure 2B). Sequencing of 10–50 thousand V3-V4 rDNA reads from *G. elata* samples was sufficient to approach saturation in endophytic bacteria richness for all samples. For all samples combined, most ASVs were represented by 8–64 reads, and others had lower or greater abundance (Figure 2C). As displayed in the Venn diagram, 282 ASVs were common between GZ and HB group. The unique ASVs among all samples collected at different groups were 674, and 119, respectively, which were 62.7% in GZ group, and 11.1% in HB group (Figure 2D). The number of endophytic bacteria ASVs of HB group less than GZ group. In addition, only 282 ASVs were common between GZ and HB group, indicating a significant difference in the community structure of endophytic bacteria between GZ and HB group.

### 3.2. Overall alpha diversity of endophytic bacteria communities in *Gastrodia elata* f.glauca tubers

The Good’s coverage threshold of 16S sequences was obtained from endophytic bacteria in *G. elata* tubers were >0.9987, indicating that it represents the true endophytic bacteria populations in the microbial community of each sample (Table 2). Higher alpha diversity index (Shannon, Simpson, Richness, Chao1 and ACE index) indicate the high diversity of endophytic bacteria community in a sample. The alpha diversity of endophytic bacteria varied among different samples for the different indices: the ASV Richness index varied from 133 to 660, with the median value of 326.16. Shannon’s diversity index varied from 1.27 to 4.31, with the median value of 2.51. ACE index varied from 136.74 to 666.64, with the median value of 345.69. Chao1 index varied from 138.25 to 671.54, with the median value of 355.66. Simpson index varied from 0.37 to 93, with the median value of 0.65. We observed the highest alpha diversity of endophytic bacteria community in GZ1 sample followed by GZ2 sample, and the lowest alpha diversity in HB3 sample. These findings indicate that the alpha diversity of the endophytic bacteria of *G. elata* was quite difference in different regions (Table 2).

**Table 2:** Alpha diversity of microbial communities in *Gastrodia elata* f.glauca tubers.

**Samples**	**Richness**	**Chao1**	**ACE**	**Shannon**	**Simpson**	**Coverage**
GZ1	660	671.54	666.64	4.31	0.93	0.9994
GZ2	356	396.60	373.25	2.73	0.68	0.9993
GZ3	371	401.36	417.18	3.20	0.79	0.9987
HB1	213	271.13	239.62	2.03	0.63	0.9992
HB2	224	255.07	240.71	1.27	0.37	0.9992
HB3	133	138.25	136.71	1.52	0.47	0.9998

### 3.3. Beta diversity analysis of the endophytic bacteria communities in *Gastrodia elata* f.glauca tubers

**Figure 3:**
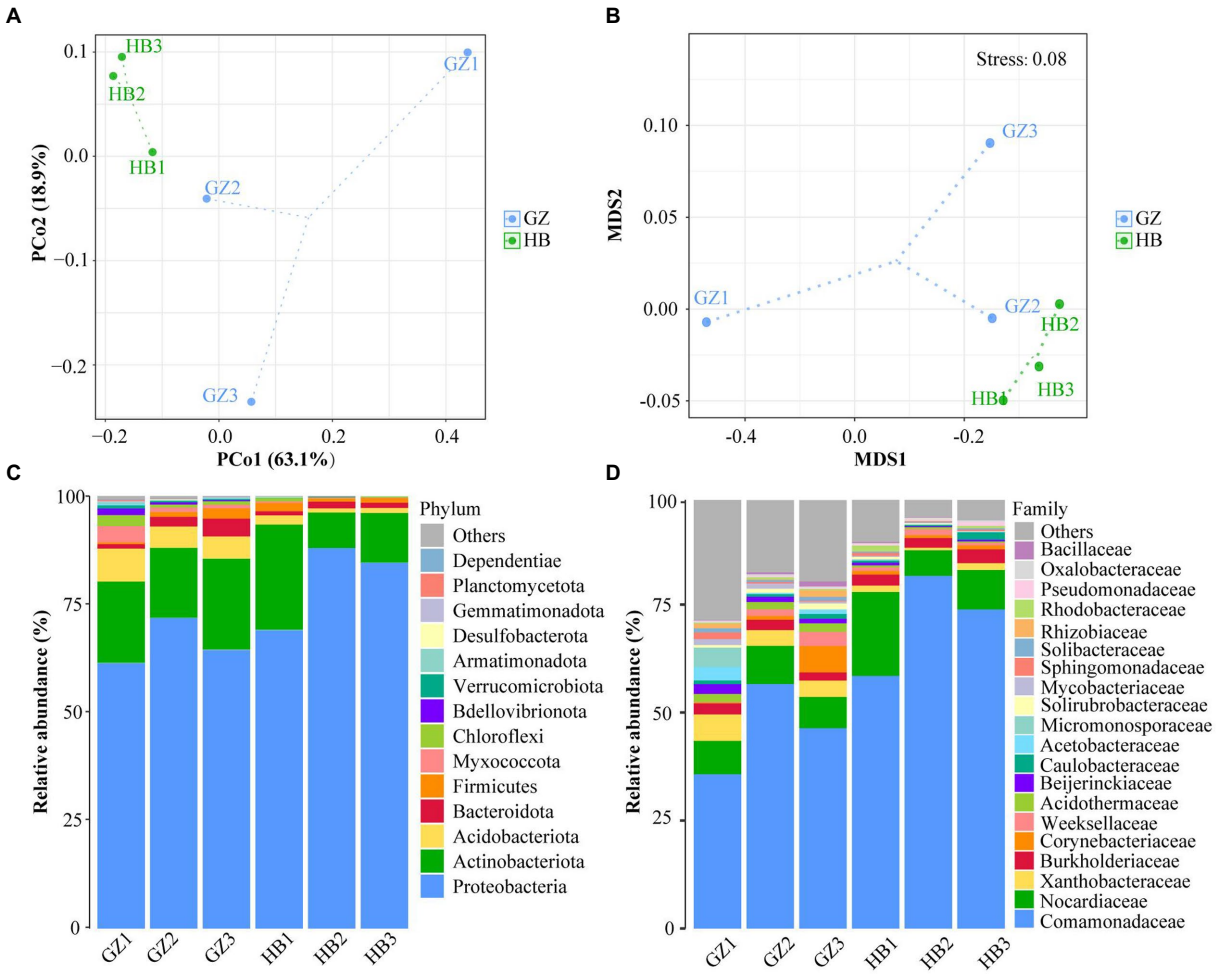
Composition of endophytic bacteria in *Gastrodia elata* f.glauca tuber samples. **(A)** Multiple sample principal coordinate analysis (PCoA) of the ASV level. **(B)** Multiple sample Non-metric multidimensional scaling (NMDS) of the ASV level. **(C)** Relative abundance of phyla in each sample. **(D)** Relative abundance of family in each sample.

To explore the Beta diversity, principal coordinate analysis (Figure 3A) and Non-metric multidimensional scaling (Figure 3B) analysis of samples of *G. elata* tubers were performed. The sum of PCoA1 and PCoA2 was 82.00% in PcoA and Stress <0.1 in NMDS, indicating a high diversity in the endophytic bacteria population structure in samples collected from *G. elata* tubers. The samples collected at HB1, HB2, and HB3 were clustered closely but were far from samples collected at GZ1, GZ2, and GZ3. These results proved the significant difference among endophytic bacteria communities in *G. elata* tubers in different regions.

The endophytic bacterial community composition of *G. elata* tubers in different regions is shown in Figure 3C at the phylum level. The results indicated that Proteobacteria (61.31%–87.95%), Actinobacteria (8.20%–18.84%) and Acdobacteriota (0.95%–7.64%) were the dominant endophytes that coexisted, in different proportions in *G. elata* tubers. Proteobacteria was the most dominant phylum in all tuber samples, with abundance ranging from 83.90%–99.87%. At the family level, every *G. elata* samples also had dominant endophytes, including mainly Comamonadaceae (35.71%–81.65%), Nocardicaece (7.24%–19.45%), Xanthobacteraceae (0.59%–6.10%) and Burkholderiaceae (1.87%–3.21%; Figure 3D).

Analysis of the two regions showed that the predominant genus (mean relative abundance ≥0.5%) of endophytic bacteria in *G. elata* tuber was *Delftia* (55.8%), followed by *Rhodococcus* (9.6%), *Corynebacterium* (1.6%), *Ralstonia* (1.4%), *Acidothermus* (1.1%), *Burkholderia* (0.9%), *Bradyrhizobium* (1.9%), *Cloacibacterium* (0.8%), *Rhizobacter* (0.7%), *Mycobacterium* (0.6%), *Brevundimonas* (0.5%), *Candidatus_Solibacter* (0.5%), *Acidibacter* (0.5%), *Roseiarcus* (0.5%). We found *Delftia* was the most dominant endophytic bacterial genus (55.8%) and *Rhodococcus* (9.6%) was the second most abundant.

### 3.4. Co-occurrence network of endophytic bacteria communities in *Gastrodia elata* f.glauca

**Figure 4:**
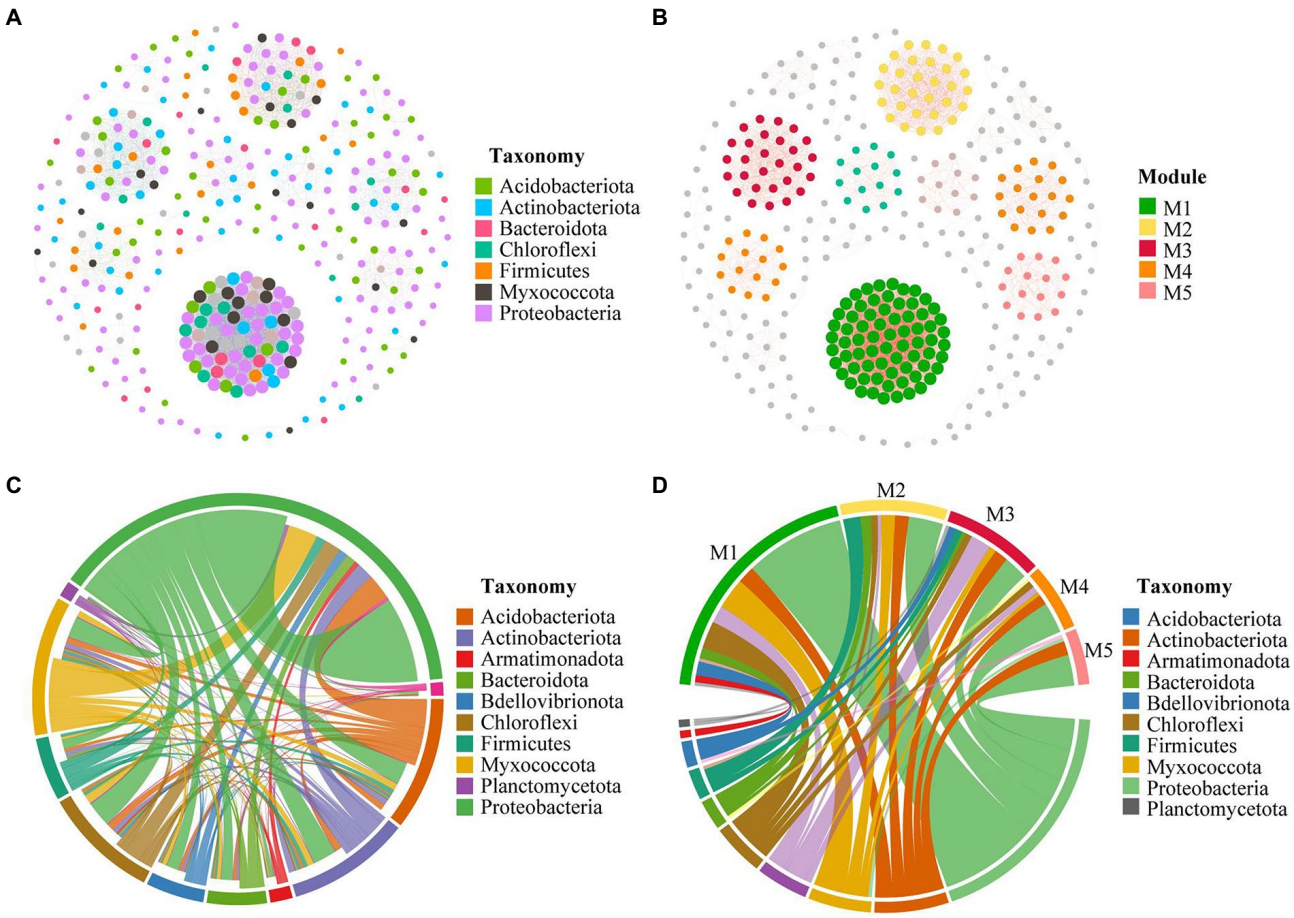
Co-occurrence network pattern. **(A)** Co-occurrence networks of endophytic bacteria communities in *G. elata* tuber; Phyla of the network as shown in different colors. **(B)** Co-occurrence networks of endophytic bacteria communities in *G. elata*; Modules of the network as shown in different colors. **(C)** Co-occurrence relationships within endophytic bacteria communities in *G. elata* tuber; phyla as shown in different colors. **(D)** Co-occurrence relationships between phyla and modules.

Co-occurrence network analysis was performed to explore potential relationships between endophytic bacteria communities in *G. elata* tuber. Modularity coefficients for all co-occurrence networks were > 0.4, indicating clear modularity. In the network, the predominant phylum (the ratio > 5%) of endophytic bacteria communities was Proteobacteria (37.43%), followed by Acidobacteriota (14.86%), Actinobacteriota (13.71%), Myxococcota (7.14%), Firmicutes (6.86%), Bacteroidota (5.71%), and Chloroflexi (5.43%; Figure 4A). In addition, there were five main modules (the ratio > 4%) in endophytic bacteria communities’ network of *G. elata* (Figure 4B).

To further investigate co-occurrence relationships within endophytic bacteria communities, we analyzed ASVs interactions among the different phyla (Figure 4C). The top six pairs with co-occurrence relationships of endophytic bacteria communities were as follows: Proteobacteria to Proteobacteria > Myxococcota to Proteobacteria > Acidobacteriota to Proteobacteria > Proteobacteria to Chloroflexi > Proteobacteria to Actinobacteriota > Proteobacteria to Bdellovibrionota. In the co-occurrence network, a higher proportion of nodes of the Proteobacteria phylum interacted with ASVs in other phyla, indicating that Proteobacteria makes the greatest contribution to the network structure. Furthermore, we found that endophyte composition was significant differences in different modules (Figure 4D). The top 5 phyla with co-occurrence relationships for each module (M1 ~ M5) were as follows: In M1, Proteobacteria > Myxococcota > Chloroflexi > Acidobacteriota > Actinobacteriota; In M2, Proteobacteria > Firmicutes > Myxococcota > Acidobacteriota > Bacteroidota; In M3, Proteobacteria > Acidobacteriota > Chloroflexi > Actinobacteriota > Myxococcota; In M4, Proteobacteria > Acidobacteriota > Chloroflexi > Actinobacteriota > Myxococcota; and M5, Proteobacteria > Acidobacteriota > Myxococcota > Bdellovibrionota > Chloroflexi. In all modules, a higher proportion of nodes of the Proteobacteria interacted with ASVs in other phyla.

## 4. Discussion

*Gastrodia elata* f.glauca is an important Chinese medicinal materials and plays a pivotal role in the regional economy in Hubei, Guizhou and other places in China (Zhan et al., 2016). According to statistics, as of 2020, the dried production of *G. elata* in China was 30,000 tons, with a total output value of about 5 billion CNY. Carrying out scientific research on endophytes in *G. elata* is important to improve the potential yield of Chinese medicinal materials, identifying the quality of herbs, and to achieve healthy planting of *G. elata*. However, the present research on G. elata is mainly focused on breeding, planting, and medicine, but there is little research on its endophytic bacteria, especially tuber endophytic bacteria. Based high-throughput sequencing methods overcome the non-culturability problem of most microbes by analyzing DNA extracted from plant endophytes (Manter et al., 2010). Plant endophytes are conventionally defined as bacteria or fungi that reside internally in plant tissues, and are in healthy plant tissues and do not harm the plant (Gaiero et al., 2013; Wang et al., 2019; Li et al., 2020). High-throughput sequencing is an efficient way to reveal the diversity of plant endophytes (Gaiero et al., 2013; Rangjaroen et al., 2019; Aswani et al., 2020).

In this study, 260,543 high-quality sequences were acquired from 6 samples of endophytic bacteria in *G. elata* and 1,265 ASVs were identified belonging to 23 phyla, 54 classes, 159 orders, 260 families, and 437 genera, which could reveal the community composition of the endophytes in *G. elata* samples. Moreover, we found that the alpha and beta diversity of the endophytic bacteria of *G. elata* was the difference in different regions. The result demonstrated that many factors, including the level of oxygen, moisture and other environmental factors, could affect diversity levels of the endophytic bacteria community (Xia et al., 2016). This found is consistent with previous studies on endophytic bacteria communities in *Camellia sinensis* and saline-alkali tolerant rice, which also found that some key factors (e.g., salt, catechins, gallic acid and anthocyanidin etc.) determine the composition of endophytic bacteria (Wu et al., 2020; Wang et al., 2021). This also explains the significant differences in the diversity and composition of endophytic bacteria of *G. elata* in different geographic regions.

In addition, we found that Proteobacteria was the most dominant phylum in all tuber samples, with abundance ranging from 83.90% to 99.87%. This found is consistent with previous studies on endophytic bacteria communities in saline-alkali tolerant rice (Wang et al., 2021). The co-occurrence networks revealed that positive correlations were the most common interactions between species, to some extent reflecting co-aggregation, cross-feeding, and co-colonization (Faust and Raes, 2012; Zhang et al., 2022). The co-occurrence network of the endophytic bacteria of *G. elata* was mainly composed of positive correlations. The results of co-occurrence network analysis also showed that a higher proportion of nodes of the Proteobacteria phylum interacted with ASVs in other clades, indicating that Proteobacteria makes the greatest contribution to the endophytic network structure (Figure 4). Overall, the main endophytic bacterial groups and community structure of different *G. elata* tuber samples collected in two regions were similar at phylum level, but the abundance was different (Figure 3C). The mainly genera are *Delftia, Rhodococcus, Corynebacterium, Ralstonia, Acidothermus, Burkholderia, Bradyrhizobium, Cloacibacterium, Rhizobacter, Mycobacterium, Brevundimonas, Candidatus_Solibacter, Acidibacter* and *Roseiarcus* (Table 3). At the same time, we found that *Delftia* and *Rhodococcus* genus were the core bacterial genus observed (55.8, 9.6%, respectively). Shockingly, *Delftia* and *Rhodococcus* were first found in *G. elata*. Recent research shows that *Delftia* is also the dominant genus of probiotic endophytes in bananas (Beltran-Garcia et al., 2021). *Delftia* can also induce resistance of whole plant to bacterial pathogens (Kurokawa et al., 2021). The role of *Delftia* and *Rhodococcus* in *G. elata* and their relationship with asparagine synthesis deserve further investigation.

**Table 3:** Summary statistics for the relative abundances of major taxonomic groups (mean ≥ 5%).

Taxonomic group	GZ1	GZ2	GZ3	HB1	HB2	HB3	Mean
Delftia	0.243	0.559	0.445	0.576	0.806	0.722	0.558
Rhodococcus	0.077	0.085	0.072	0.192	0.058	0.091	0.096
Corynebacterium	0.002	0.009	0.061	0.009	0.007	0.009	0.016
Ralstonia	0.006	0.014	0.011	0.016	0.013	0.022	0.014
Acidothermus	0.019	0.017	0.020	0.005	0.004	0.003	0.011
Burkholderia	0.012	0.009	0.007	0.009	0.010	0.010	0.009
Bradyrhizobium	0.024	0.008	0.011	0.005	0.002	0.003	0.009
Cloacibacterium	0.001	0.009	0.018	0.005	0.011	0.003	0.008
Rhizobacter	0.041	0.000	0.000	0.000	0.001	0.000	0.007
Mycobacterium	0.014	0.012	0.004	0.002	0.002	0.002	0.006
Brevundimonas	0.001	0.003	0.005	0.004	0.001	0.017	0.005
Candidatus_Solibacter	0.009	0.005	0.009	0.003	0.001	0.003	0.005
Acidibacter	0.008	0.011	0.005	0.002	0.001	0.004	0.005
Roseiarcus	0.005	0.008	0.010	0.004	0.001	0.001	0.005

## 5. Conclusion

This study first investigated the endophytes of the *G. elata* tubers in the Guizhou and Hubei region using the high-throughput sequencing methodology. The results show that endophytes communities exhibited significant geographic variations. The alpha and beta diversity of endophytes in *G. elata* was higher in Guizhou and declined in Hubei. Moreover, At the phylum level, the dominant phyla were Proteobacteria, Actinobacteria and Acdobacteriota. At the family level, the dominant family were Comamonadaceae, Nocardicaece, Xanthobacteraceae and Burkholderiaceae. At the genus level, *Delftia* and *Rhodococcus* were represented the core microbiota in *G. elata* tuber. The coexistence of core bacteriome and tubers, indicates that should be beneficial to the growth and health of *G. elata* plants. The physiological and ecological functions of *Delftia* and *Rhodococcus* and their relationship with active components of *G. elata* are worthy of further study.

## Data availability statement

The data presented in the study are deposited in the National Genomics Data Center repository, accession number CRA009458.

## Author contributions

HZ, PZ, JD, and JG designed and participated in all experimental procedures, performed data analysis, and drafted the manuscript. JD participated in the samples collection and preparation. HZ, PZ, JQ, JD, and JG supervised the study and critically revised the manuscript. All authors have read and agreed to the published version of the manuscript.

## Funding

This study was supported by the Hubei Provincial Administration of Traditional Chinese Medicine Research Project of Traditional Chinese Medicine (Project No. ZY2023F030).

## Conflict of interest

The authors declare that the research was conducted in the absence of any commercial or financial relationships that could be construed as a potential conflict of interest.

## Publisher’s note

All claims expressed in this article are solely those of the authors and do not necessarily represent those of their affiliated organizations, or those of the publisher, the editors and the reviewers. Any product that may be evaluated in this article, or claim that may be made by its manufacturer, is not guaranteed or endorsed by the publisher.

## Abbreviations

*G. elata*: *Gastrodia elata Bl. form.* Glauca S. Chow
ASVs: amplicon sequence variants
PCoA: principal coordinate analysis
NMDS: non-metric multidimensional scaling
